# A Pediatric Epidural Catheter Fracture Suspected to Be Caused by a Glue

**DOI:** 10.7759/cureus.60524

**Published:** 2024-05-17

**Authors:** Masahiro Yagihara, Aki Uemura, Chiharu Wakuda, Sho Sugimura, Yoshiki Nakajima

**Affiliations:** 1 Department of Anesthesiology and Intensive Care, Hamamatsu University School of Medicine, Hamamatsu, JPN; 2 Department of Anesthesiology, Anshin Hospital, Kobe, JPN

**Keywords:** ethyl-2-cyanoacrylate, surgical removal, ultrasound examination of the spine, microscopic examination, fractured pediatric epidural catheter

## Abstract

A 65-day-old baby boy underwent the Kasai procedure under general and epidural anesthesia. The epidural catheter was inserted between the T11 and T12 vertebrae under general anesthesia, and secured with sterile tape, ethyl-2-cyanoacrylate glue, and film. Intra- and postoperative epidural analgesia was effective and there was no leakage around the insertion site. On the third day post-surgery, we tried to remove the catheter but discovered it was fractured 67mm from the tip. During the ultrasound examination, we observed a hyper-echoic structure located between the laminae of T11/T12. The pediatric orthopedic surgeon recommended removing the catheter to avoid long-term neurological sequelae of leaving the catheter, such as infection, fibrosis, migration, and irritation of neural tissues. It was surgically removed uneventfully on postoperative day 4. We requested the manufacturer to inspect the cross-section of the catheter under a microscope. The cross-section showed that 20% of the area had undergone tearing due to traction, while the remaining 80% was cracked. We also requested the manufacturer simulation after that. The same catheter, fixed on the polyolefin resin plate instead of skin with the same tape and glue, was easily fractured after three days. It is suspected that using ethyl-2-cyanoacrylate glue caused the catheter to fracture. When using glue containing ethyl-2-cyanoacrylate for pediatric epidural catheter fixation, special care is advised.

## Introduction

A fractured epidural catheter is a recognized complication in both adults, infants, and children. The prevalence of this complication was 0.002-0.055% in adults [1,2]. However, there are limited case reports and guidelines on this complication in infants and children, which makes it difficult to determine whether the remnant of the catheter should be removed or left in place [3].

Typically, epidural catheters fracture when the catheter is inserted or removed. However, here we found the catheter fractured without applying traction but after the plastic film covering the catheter was removed. Based on conducting microscopic examinations and simulation tests, we suspect that the glue we used for fixation on an infant may be the actual cause of the problem.

## Case presentation

We obtained written consent from the parents before publishing this information. Approval of the Ethics Committee for Life Science and Medical Research, Hamamatsu University School of Medicine (# 81-003) was obtained.

A 65-day-old boy underwent a Kasai operation for congenital biliary atresia. He measured 54.6 cm in height and weighed 5.1 kg. For the surgery, we used combined general and epidural anesthesia. To insert the catheter, we used a 19 G Tuohy needle (35 mm in length) and a polyethylene epidural catheter, with a diameter of 0.6 mm, from Hakko Co., Ltd. in Japan. We inserted the catheter between the T11 and T12 vertebrae. Using the micro-drip infusion method [4], we successfully performed the epidural puncture without any issues, and the catheter was smoothly inserted without any resistance. The distance between the skin and the epidural space was 12 mm.

To ensure that the catheter remained in place, it was fixed over the skin at 65 mm from the tip, with sterilized tape (Steri-Strip, 3M, St. Paul, USA). Ethyl-2-cyanoacrylates (ECA) glue (Aron Alpha, West Jefferson, USA) was applied, unintentionally more than the usual amount used, to the puncture site and then covered with a film. Intra- and postoperative epidural analgesia was administered through the catheter, with a 0.2% levobupivacaine infusion of 1 mL per hour. Following a six-hour and 40-minute surgery, the total blood loss during the procedure was 56 mL. The epidural analgesia was effective and there was no leakage around the insertion site during the postoperative period.

**Figure 1:**
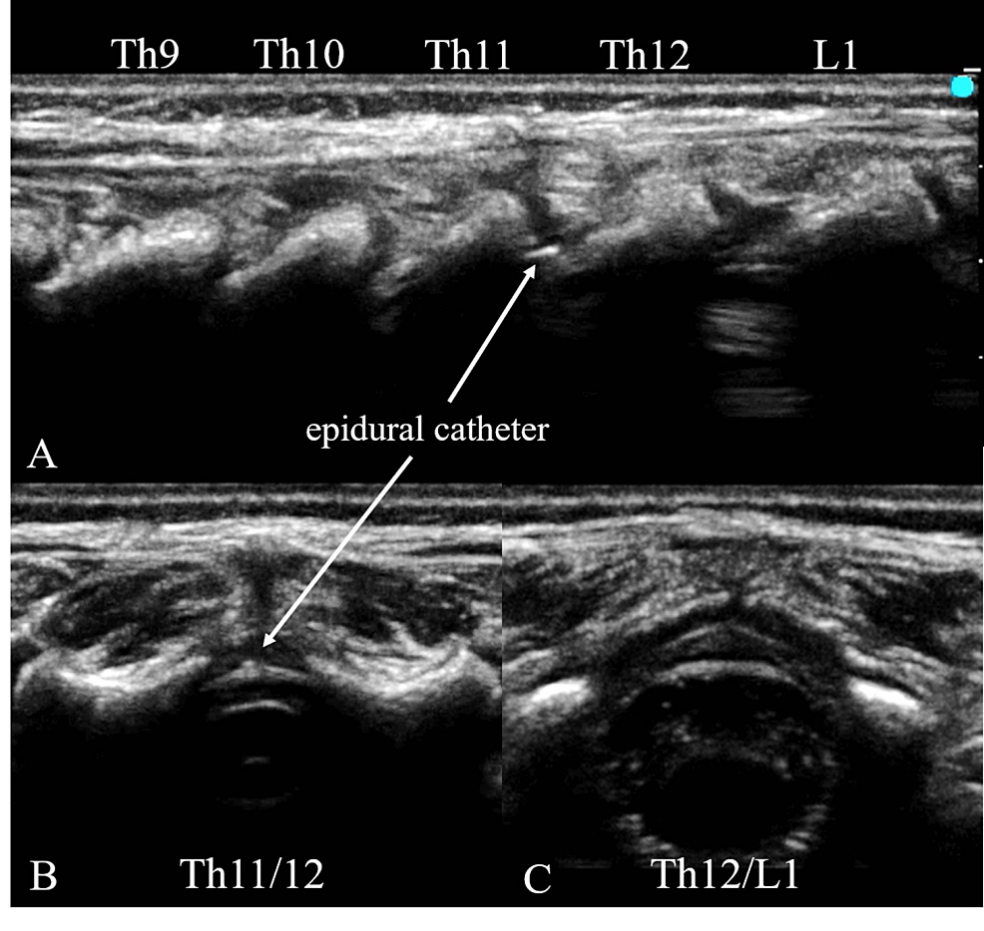
Ultrasound examination of the spine A: Sagittal view of the spine. The epidural catheter, as a hyper-echoic structure (arrow), was detected at Th11/12. B: Axial view of Th11/12. The remainder of the catheter, as a hyper-echoic dot, was detected in the epidural space. C: Axial view of Th12/L1. Nothing was detected in the epidural space.

**Figure 2:**
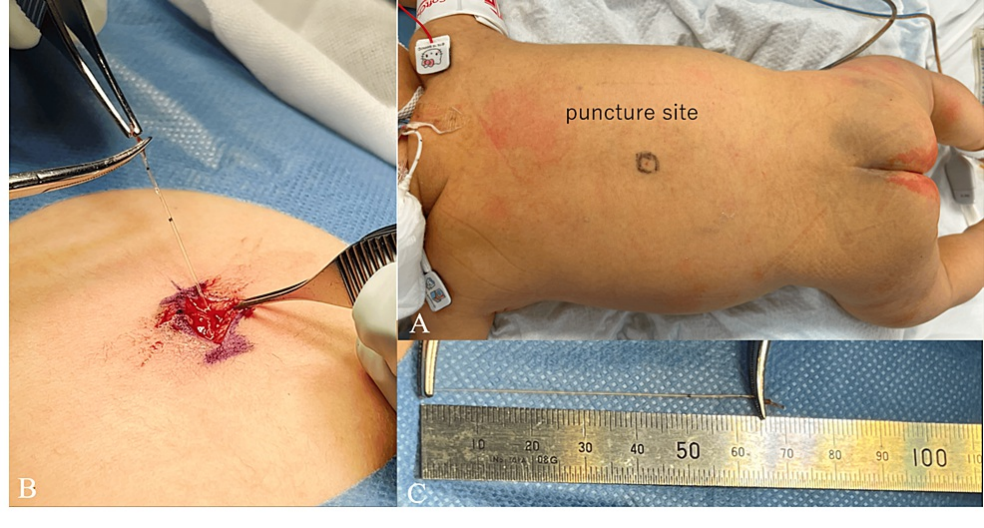
Surgical removal of the remaining epidural catheter A: The puncture site still remained immediately before surgery. B: The catheter remnant was discovered soon after the skin incision. C: All of the remaining catheter (67mm) was removed uneventfully.

On the third day post-surgery, we attempted to remove the epidural catheter, only to discover that it had snapped 67 mm from the tip and bonded to the sterilized tape. It happened soon after removing the film without any catheter traction. We were unable to palpate the remnant of the catheter fragment at the puncture site. An ultrasound revealed a hyper-echoic structure between the lamina of T11/12 (Figure 1). We consulted with a pediatric orthopedic surgeon, who advised the immediate removal of the catheter to avoid long-term neurological sequelae of leaving the catheter, such as infection, fibrosis, migration, and irritation of neural tissues. In addition, we estimated the remnant was located in the superficial tissue, removal via surgery may be a minimally invasive option. On the fourth day following the procedure, the orthopedic surgeon successfully located and extracted the catheter (Figure 2). The patient has not had any neurological sequelae since the removal.

**Figure 3:**
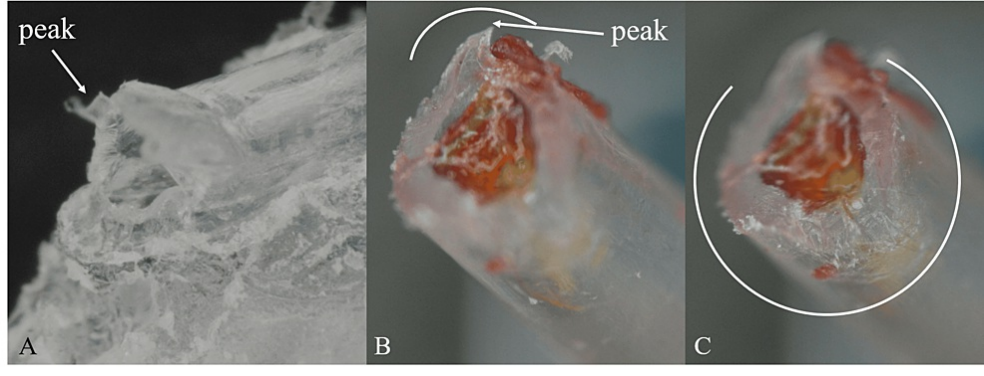
The microscopic examination of the fractured epidural catheter A: The cross-section of the fractured epidural catheter bonded to the tape and film. There is a peak on the opposite side of the film. B: The cross-section of the removed epidural catheter. Around 20% of it (white curve) is torn off due to traction and a peak appears (white arrow). C: The cross-section of the removed epidural catheter. Around 80% of it (white curve) is cracked. This is demonstrated by its roughness and its diameter not becoming thinner.

We requested that the manufacturer inspect the cross-section of the fractured catheter using a microscope and investigate the cause of the fracture. The microscopic examination confirmed that the catheter had broken off by deterioration rather than just torn off by traction, as demonstrated by a demarcation. Although 20% of the catheter was torn by traction, its diameter remained unchanged. The cross-section was too rough and not flat, causing 80% of it to crack instead of being cleanly cut off (Figure 3). We suspect that the main cause of the fracture was due to the deterioration of the catheter. Something may have interacted with the polyethylene composition of the epidural catheter until postoperative day 3 and fractured when the film was removed. The exit of the epidural catheter, 65 mm from the tip as it emerges from the skin, and the fractured point, 67 mm from the tip, were almost in the same location. This has led us to suspect that the ECA glue used for reinforcement caused the deterioration of the catheter.

**Figure 4:**
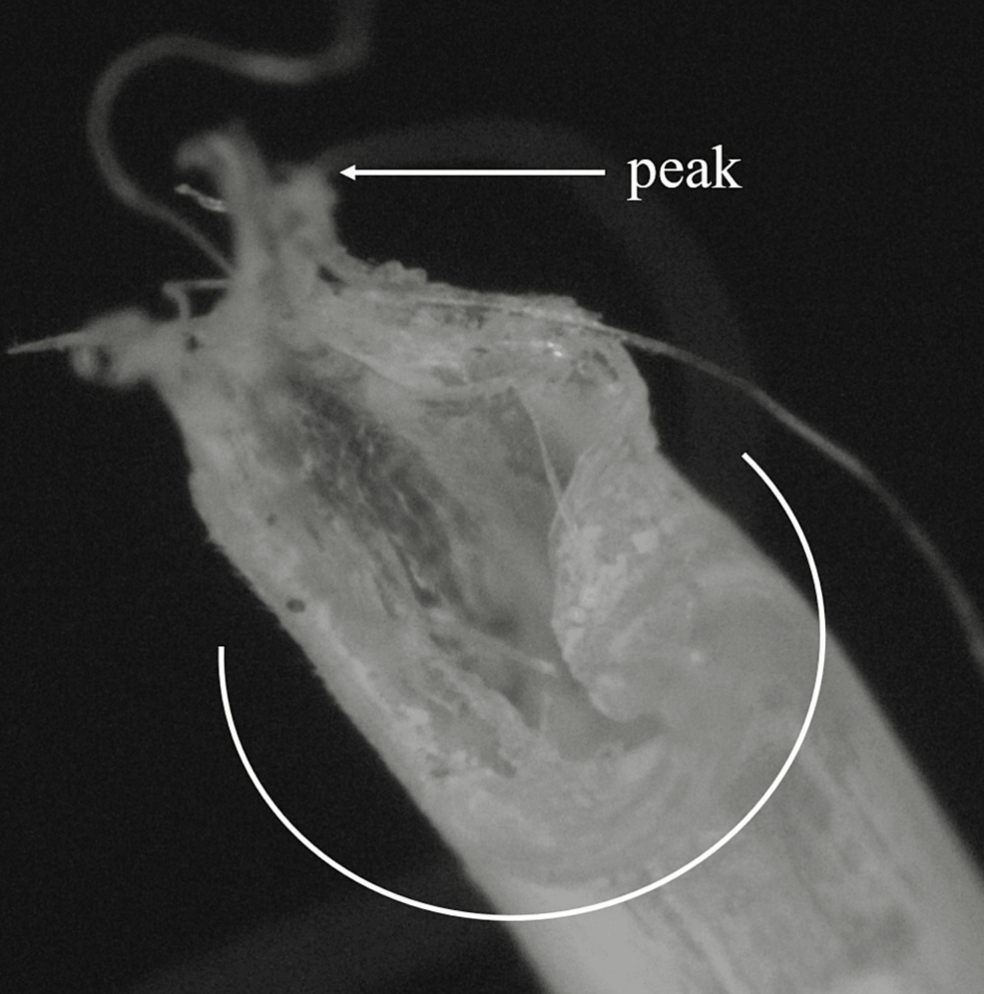
The cross-section of the catheter simulated with the same tape, film, and glue under the microscope Approximately half of the catheter (white curve) is fractured and the other is torn off on account of traction, with a peak appearing.

We also requested the manufacturer to replicate the same scenario. During the replication trial, using the same tape and glue as on the skin, the catheter was fixed on the polyolefin resin plate. Then, after being left for three days, it broke while the catheter was being removed from the plate by traction. The cross-section of the simulated catheter was identical to the remnant one (Figure 4). The manufacturers had never tested their catheters with this ECA glue before and discovered it could cause the catheter to deteriorate and break within three days.

## Discussion

Remnants of the catheter fragments post-epidural can cause severe complications such as hematomas, abscesses, and nerve injuries. The optimal diagnostic and treatment strategy for a remnant of the epidural catheter is still controversial, especially in infants and children (Table 1) [3,5-12]. The causes of catheter fractures were previously attributed to operator errors during insertion or withdrawal, pressure and/or extension on the catheter while in the body, and difficulty removing it due to migration, pinching, and knotting [3,5-12].

**Table 1:** Review of cases with retained epidural catheters in infants and children and their management CT: computed tomography; MRI: magnetic resonance imaging; US: ultrasound

Report	Age	Weight (kg)	Gender	Catheter type	Puncture level	Investigation category	Epidural catheter problem	Neurological sequelae	Problem causes	Treatment	Follow-up period
Armerdi et al., 1992 [5]	1y9m	10	Male	Kendol Safe Trak 20G Radiopaque	Caudal	X-ray and CT	Broken at 7.8cm from the distal tip	None	Cut by needle during tunneling	Surgery subcutaneous	5 days
Lenox et al., 1995 [6]	1y11m	10	Male	Baxter 20G Radiopque	Caudal	X-ray and CT	Broken at 8cm from the distal tip	None	Twisting or crimping	Surgery subcutaneous	Not mentioned
Bercowitz et al., 2005 [7]	5w	4.9	Male	Portex 20G	Caudal	Contrast X-ray	Migrated to the paravertebral space	None	Impeded by nerve roots or dorsomedian septa suspected	Removed	None
Joselyn et al., 2014 [8]	2d	2	Not mentioned	Arrow FlexTip Plus 20G Radiopaque	Caudal	None	Knotted at the distal tip	None	Not mentioned	Traction after 1h heat pack	Not mentioned
Eu et al., 2017 [3]	2y	Not mentioned	Female	B-Braun Perifix One Paed 20G Radiopaque	Caudal	CT and MRI	Broken at 6cm from the distal tip	None	Twisting and crimping	Observation	1 year
Sardana et al., 2017 [9]	10y	Not mentioned	Female	Portex Radiopaque	L1/2	X-ray	Broken at 5cm from the distal tip	None	Withdrawing through the needle	Observation	15 months
Ravishankar, 2017 [10]	5m	5	Male	B-Braun 24G	L2/3	CT	Knotted and looped at 9cm from the distal tip	None	Too much insertion to the epidural space	Surgery laminectomy	Not mentioned
Visoiu et al., 2019 [11]	5m	3.56	Male	B-Braun Perifix 20G Radiopaque	Paravertebral	US	Migrated from the paravertebral space to the epidural space	None	Not visualize the catheter tip by ultrasound	Removed	None
Smedile et al., 2020 [12]	5m	5.27	Female	B-Braun Perifix One Paed 24G Radiopaque	L4/5	CT	Broken at 6mm from the distal tip	None	Stretched beyond tensile strength under the tip pinching	Observation	6 months

We suspect that ECA glue is the primary cause of the issue for several reasons. Firstly, the procedure of insertion was carried out smoothly, without any resistance or errors made by the operator. Secondly, as the catheter was being removed from the patient's body, it fractured at the same point where the glue was applied. Thirdly, the catheter's cross-section under microscopic examination revealed that it had been fragile. Most areas of the cross-section were rough (not flat), thus, excluding the possibility of being cut off by the Tuohy needle. We conducted a search for literature but could not find any case reports indicating that ECA glue had caused any damage or deterioration to the epidural catheter.

**Figure 5:**
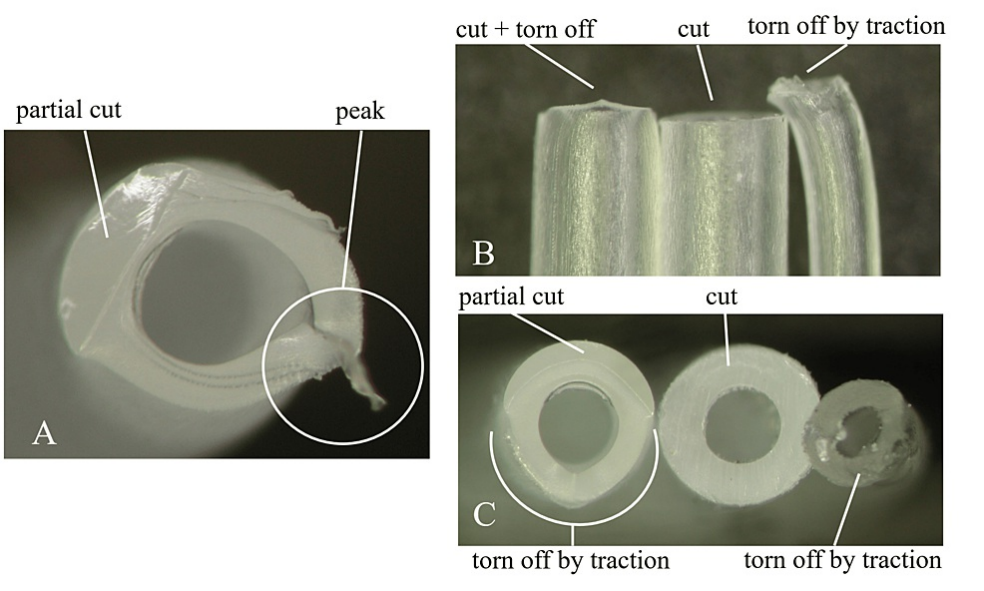
The difference of the cross-section by the causes of the fracture A: Under the microscope, the cross-section of the catheter is partially cut and torn off owing to traction. The cut area is smooth and there is a peak on the opposite side of the cut. The diameter becomes slightly thinner at the area of traction. B: The side view of three catheters (cut+torn off, cut, torn off). The diameter of the torn off catheter becomes obviously thinner. C: The cross-section of the catheters from picture B.

The manufacturer's analysis revealed that the catheter, fixed with ECA glue, had fractured. If a catheter is forcefully extended and breaks, it may result in the catheter wall becoming thinner. As a result of the tear by traction, “the peak” is observed at the area of occurrence (Figure 5).

The epidural catheter used for pediatric patients is thinner, which increases the risk of drug fluid leakage when it is placed a short distance from the skin to the epidural space. ECA, the same glue we used for our infant, was also used uneventfully to secure the epidural catheter during the cesarean section [13]. We used the glue to avoid peri-catheter leakage in pediatric epidural anesthesia. Nevertheless, it may not be safe for the pediatric epidural catheter due to its low tolerance (with an outer diameter of 0.6 mm).

ECAs have been widely used as surgical adhesives because of their biocompatibility and high reactivity under wet conditions. However, the use of ECAs for epidural catheter fixation is not common practice. And it has been found that the toxicity decreased as the alkyl chain length increased. As a result, cyanoacrylate monomers with short alkoxy carbonyl side chains, such as methyl 2-cyanoacrylate and ECA are no longer used [14]. Cyanoacrylates also cause long-lasting inflammatory reactions in tissues, but by increasing the chain length of this polymer it can be less immunogenic [15]. In a narrative literature review of peripheral nerve catheter securement, 2-octyl cyanoacrylates (OCA) glue is widely used for reducing rates of dislodgement or leakage [16]. Combined OCA and n-butyl 2 cyanoacrylate (NBCA) glue was reported to be a good candidate as a sealant of the 4 Fr or 5 Fr PI-catheters exit site [17].

We started to use ECA as the adhesive for the epidural catheter in 2018. This is the first time we have experienced a fracture of the catheter. The reason why the fracture occurred at this time was not clear. However, more glue was unintentionally applied than usual, which may have partly contributed to the damage.

## Conclusions

The analysis of the catheter cross-section and additional simulation indicated that the medical glue might have caused fragility in the catheter. In view of the complication stated, we suggest that, in order to avoid any damage to the epidural catheter in infants and children, ECA glues should not be used henceforth.
